# Supplementary material for: Content-rich biological network constructed by mining PubMed abstracts
Source: BMC Bioinformatics. 2004 Oct 8;5:147. doi: 10.1186/1471-2105-5-147 (PMC528731; doi:10.1186/1471-2105-5-147)
Supplement: Additional File 2 — The original results of the above study (non-essential files are deleted to keep the file size under the limit set by BMC bioinformatics). [file 1471-2105-5-147-S2.bz2 › chilibotAdditionalFile2/dip20/724ID10920254E18072/html/ACRA_ACRB.html]

 


 **ACRA** and **ACRB** 
  
Found 19 abstracts in PubMed, retrieved 19.  
 

 What does Google say? 
 PDF only 
| .edu only 

---

**Interactive relationship** (e.g. stimulation, inhibition, etc)

**Neutral relationship**- We conclude that  **AcrA**  exists as a complex with  **AcrB**  on the periplasmic surface of the inner membrane after removal of the signal sequence.  Ref: 10920254 J Biochem Tokyo , 2000
- In this oligomeric form,  **AcrA**  interacts specifically with  **AcrB**  transporter independently of substrate and TolC.  Ref: 10894736 J Bacteriol, 2000
- After SDS PAGE, Western blotting was performed with anti TetBCt antiserum, resulting in the appearance of a 40 kDa band, indicating that  **AcrA**  co precipitated with  **AcrB** .  Ref: 10920254 J Biochem Tokyo , 2000
- The coordinated operation of the inner membrane transporter  **AcrB**  and outer membrane channel TolC is thought to be mediated by  **AcrA** .  Ref: 9878415 J Mol Biol, 1999
- **AcrA**  was thought to be localized in the periplasmic space, and to be linked to  **AcrB**  and TolC.  Ref: 10920254 J Biochem Tokyo , 2000
- Molecular construction of a multidrug exporter system, AcrAB molecular interaction between  **AcrA**  and  **AcrB** , and cleavage of the N terminal signal sequence of  **AcrA** .  Ref: 10920254 J Biochem Tokyo , 2000

**Non-interactive relationship** (e.g. studied together, co-existance, homology, etc.)

- The tripartite  **AcrA**   **AcrB**  TolC system is the major efflux pump of the nosocomial pathogen Enterobacter aerogenes.  Ref: 12651015 J Chromatogr B Analyt Technol Biomed Life Sci, 2003
- In order to reconstitute the  **AcrA**   **AcrB**  TolC system of E. aerogenes in artificial membranes, we overexpressed and purified the three proteins.  Ref: 12651015 J Chromatogr B Analyt Technol Biomed Life Sci, 2003
- **AcrB**  is a proton motive force dependent transporter located in the inner membrane, and  **AcrA**  and TolC are accessory proteins located in the periplasm and the outer membrane, respectively.  Ref: 10894736 J Bacteriol, 2000
- Strains defective in one of the genes,  **acrA** ,  **acrB** , or tolC, were remarkably sensitive to organic solvents.  Ref: 9783171 Extremophiles, 1998
- The AcrAB system of Escherichia coli is a multidrug efflux system composed of an RND type transporter  **AcrB**  and a periplasmic accessory protein  **AcrA** , and pumps out a wide variety of lipophilic and amphiphilic inhibitors directly into the medium, presumably through the TolC outer membrane channel.  Ref: 11321576 J Mol Microbiol Biotechnol, 2001
- These results cannot be explained by simply assuming that AcrD, not hitherto known to interact with  **AcrA** , acquired this ability by the introduction of the loop regions of  **AcrB** , because i both AcrD and  **AcrA**  were found, in this study, to be required for the efflux of amphiphilic substrates, and ii chemical cross linking in intact cells efficiently produced complexes between AcrD and  **AcrA** .  Ref: 12426336 J Bacteriol, 2002
- Overexpression and purification of the three components of the Enterobacter aerogenes  **AcrA**   **AcrB**  TolC multidrug efflux pump.  Ref: 12651015 J Chromatogr B Analyt Technol Biomed Life Sci, 2003
- Genes  **acrA**  and  **acrB**  encode a stress induced efflux system of Escherichia coli.  Ref: 7651136 Mol Microbiol, 1995
- Defined mutations of  **acrA**  or  **acrB**  formerly acrE genes increased the susceptibility of Escherichia coli to a range of small inhibitor molecules.  Ref: 7651136 Mol Microbiol, 1995
- Together with  **AcrA**  and TolC,  **AcrB**  forms a proton motive force dependent efflux pump of the resistance nodulation cell division RND transporter superfamily and is responsible for resistance towards many common antibiotics such as ciprofloxacin and novobiocin.  Ref: 12351840 Acta Crystallogr D Biol Crystallogr, 2002
- TolC is an outer membrane channel, and  **AcrA**  is an elongated lipoprotein that is hypothesized to span the periplasm and coordinate efflux of such substrates by  **AcrB**  and TolC.  Ref: 12426336 J Bacteriol, 2002
- Porphyromonas gingivalis, a gram negative obligate anaerobe, contains two homologs of an Escherichia coli resistance nodulation cell division type multidrug exporter gene,  **acrB** , in putative operons, together with homologs of membrane fusion protein gene  **acrA**  and outer membrane channel gene tolC.  Ref: 12234854 Antimicrob Agents Chemother, 2002
- **AcrA**  is a trimeric periplasmic lipoprotein anchored in the inner membrane,  **AcrB**  is an inner membrane transporter and TolC is a trimeric outer membrane channel.  Ref: 12651015 J Chromatogr B Analyt Technol Biomed Life Sci, 2003
- We identified the genes encoding the  **AcrA**   **AcrB**  TolC efflux pump in Enterobacter aerogenes and constructed acrAB and tolC mutants from a multidrug resistant isolate.  Ref: 12121946 Antimicrob Agents Chemother, 2002
- A mutant plasmid encoding both the  **AcrA**  TetBCt fusion protein and the  **AcrB**  His fusion protein was constructed.  Ref: 10920254 J Biochem Tokyo , 2000
- The E. aerogenes  **acrA** ,  **acrB**  and tolC open reading frames were individually inserted in the expression vector pET24a, in frame with a sequence coding a C terminal hexahistidine tag to allow purification by INAC Immobilized Nickel Affinity Chromatography.  Ref: 12651015 J Chromatogr B Analyt Technol Biomed Life Sci, 2003
- Cross linked complex between oligomeric periplasmic lipoprotein  **AcrA**  and the inner membrane associated multidrug efflux pump  **AcrB**  from Escherichia coli.  Ref: 10894736 J Bacteriol, 2000
